# Tungsten Carbide/Tungsten Oxide Catalysts for Efficient Electrocatalytic Hydrogen Evolution

**DOI:** 10.3390/molecules30010084

**Published:** 2024-12-29

**Authors:** Jian Ouyang, Yu Sun, Yiqiong Zhang, Juzhe Liu, Xin Bo, Zenglin Wang

**Affiliations:** 1 Shenzhen Kohodo Hydrogen Energy Co., Ltd., Shenzhen 518109, China; ouyangjian@kohodo.cn; 2 Key Laboratory of Applied Surface and Colloid Chemistry, Ministry of Education, School of Chemistry and Chemical Engineering, Shaanxi Normal University, Xi’an 710119, China; 3 College of Materials Science and Engineering, Changsha University of Science & Technology, Changsha 410114, China; 4 Key Laboratory of Resources and Environmental Systems Optimization, Ministry of Education, College of Environmental Science and Engineering, North China Electric Power University, Beijing 102206, China

**Keywords:** tungsten carbide, tungsten oxide, heterogeneous electrocatalysts, hydrogen evolution reaction

## Abstract

Catalyzing hydrogen evolution reaction (HER) is a key process in high-efficiency proton exchange membrane water electrolysis (PEMWE) devices. To replace the use of Pt-based HER catalyst, tungsten carbide (W_2_C) is one of the most promising non-noble-metal-based catalysts with low cost, replicable catalytic performance, and durability. However, the preparation access to scalable production of W_2_C catalysts is inevitable. Herein, we introduced a facile protocol to achieve the tungsten carbide species by plasma treatment under a CH_4_ atmosphere from the WO_3_ precursor. Moreover, the heterogeneous structure of the tungsten carbide/tungsten oxide nanosheets further enhances the catalytic activity for HER with the enlarged specific surface area and the synergism on the interfaces. The prepared tungsten carbide/tungsten oxide heterostructure nanosheets (WO_3-x_-850-P) exhibit exceptional HER catalytic activity and stable longevity in acid electrolytes. This work provides a facile and effective method to construct high-performance and non-precious-metal-based electrocatalysts for industrial-scale water electrolysis.

## 1. Introduction

With the rapid consumption of fossil energy, hydrogen energy is regarded as one of the most promising candidates for an energy carrier, dealing with the predicament of energy shortage and environmental deterioration. Hydrogen energy’s properties of high energy density and being renewable and pollution-free have become the focus of people’s attention. It is expected to replace non-renewable fossil energy and to be used for fuel cells and raw materials preparation of various organic molecular compounds [1,2,3,4]. Hydrogen can be produced on a large scale by water electrolysis, including green hydrogen through the electrochemical process driven by solar, wind, hydropower, etc. However, the hydrogen evolution reaction (HER) in the conventional alkaline water electrolysis (AWE) by using the nickel cathode presents low reaction activity and large hydrogen evolution overpotential, resulting in the increase in the voltage of the electrolyze and further consuming the unnecessary energy input. Therefore, it is urgent to develop an advanced HER electrocatalyst to achieve massive hydrogen production from water electrolysis [5,6,7,8]. At present, the benchmark catalysts are mainly noble metal-based materials consisting of Pt, Pd, Ir, and Rh (platinum group metal, PGM), whose catalytic activity is close to the ideal value (almost no overpotential for HER), causing fast reaction kinetics, since these catalysts can effectively optimize the kinetic barrier of H* intermediate formation. However, the scarcity of the noble metal resource severely increases the cost of the electrolysis devices, which is inappropriate for large-scale commercial applications [9,10,11,12]. Therefore, it is of great importance to develop a noble-metal-free catalyst with high activity for HER [13,14,15].

Transition metal-based materials such as alloys, chorionic compounds, carbides, nitrides, borides, and phosphates have attracted extensive attention. Compared to other transition metal materials, tungsten carbides (WC and W_2_C) have a wider d-band, which can modify the local interfacial electronic structure, which is similar to that of PGM in a wide pH compatibility range when they are used as the electrocatalysts, resulting in promising catalytic performance [16,17,18,19]. Furthermore, the appropriate work function of 5.0 eV, outstanding stability, and strong resistance against catalytic poisoning with specific oxygen adsorption make WC material a promising substitute for noble metal catalysts [19,20]. However, WC has low HER activity due to the high binding strength of W and H, which severely inhibits hydrogen desorption. By changing the chemical environment and coordination structure of WC, the binding strength of W-H [21,22,23,24] can be sufficiently optimized.

On the other hand, the tungsten carbide is always fabricated via a solid–gas reaction under an extremely high-temperature carbonization range (1400–1700 °C) in a particular vacuum or hydrogen atmosphere [25,26], which is quite a harsh requirement for massive production and low yielding. In addition, the tungsten carbide materials obtained by direct carbonization are often lumpy materials or nanoparticles with poor crystallinity and impurities, which have a negative impact on the performance of HER. To address this challenge, common strategies are used to accomplish the morphology control, composition, and electronic regulation so as to boost the HER performance, including multi-step pyrolysis, solvothermal method, solid-state synthesis strategy, etc. [17,18,19]. For example, Wu et al. prepared mesoporous WC nanoplates with unique configurations and electron distribution by combining a hydrothermal reaction and a gas–solid carburizing process. In the acidic solution, the initial overpotential of HER was 63 mV, the slope of Tafel was 58 mV·dec^−1^, and the catalytic performance did not decrease significantly after a long cycle, showing good HER activity and stability [27].

Meanwhile, using the excess gas (containing the carbon content) precursor also carries the risk of surface carbonization, cooking the surficial active sites [27,28,29,30,31,32,33]. For example, Wang et al. fabricated in situ stable-binding, nitrogen-doped WC/W (WC-N/W) heterostructures [34]. The WC-N/W electrode showed excellent HER performance in both acidic and alkaline media, with the lowest overpotential of 87 and 104 mV and Tafel slopes of 44.9 and 62.2 mV dec^−1^, respectively. It also maintains a stable duration at a high current density of 1000 mA cm^−2^. The superior performance is attributed to the super hydrophilic and structural characteristics of the ceramic membrane, as well as the effect of the WC-N/W heterogeneous interface and formation of the pyri-N, pyrr-N, grap-N species, among which pyri-N and pyrr-N have been proven to promote hydrogen generation in HER systems, optimizing the adsorption energy with H* to accelerate the charge transfer process [35,36]. However, the annealing temperature for this material rises as high as 1600 °C. Sun et al. synthesized N-doped carbon-encapsulated bimetallic electrocatalyst (Co/WC@NC) using polyvinylamine (PEI) as carbon source and the polyoxometalate (POM) and cobalt ions as metal precursors [37]. In both acidic and alkaline media, Co/WC@NC showed good HER activity with 158 mV and 178 mV overpotentials at 10 mA cm^−2^, respectively. In addition, it displays excellent stability over 24 h. The performance improvement is due to the significant increase in WC’s state density as the induction by Co charge delocalization, leading to moderate H_2_O binding energy and weakened H adsorption. Moreover, an N-doped carbon layer can improve the electrical conductivity and protect Co and WC nanoparticles from corrosion. However, the use of polyvinylamine reagents is highly toxic, which seriously limits its large-scale synthesis and application.

As we know, plasma consists of atoms, molecules, ions, and free radicals with equal amounts of positive and negative charges. The excitation of plasma is mainly obtained by the ionization of gas molecules when sufficient energy is applied to them. Studies have shown that in the synthesis of materials, plasma can usually play a role in promoting the reaction or directly use the plasma of special gases to achieve the transformation of materials [38,39]. The conversion from metal oxides to metal carbides can be achieved by plasma treatment under CH_4_ atmospheres. In the application of plasma to material modification, the most common application is to use its etching effect to promote the exposure of more edge catalytic active sites on the surface of the material and realize the doping of some heteroatoms or the construction of material defects, so as to improve the electrocatalytic activity.

In this work, we prepared the nano-sized tungsten carbide/tungsten oxide catalysts through a more convenient plasma strategy and efficient carburizing method. Plasma technology is one of the most commonly used and effective strategies for the surface modification and defect construction of nanomaterials. As the fourth state of matter, plasma is composed of atoms, molecules, ions, and free radicals with equal amounts of positive and negative charges. Generally speaking, the excitation of plasma is mainly obtained by ionizing gas molecules when sufficient energy is applied to them so that surface modification, construction defects, doping, and so on can be quickly achieved. Specifically, we use plasma under methane atmosphere to process tungsten oxide nanosheets so that tungsten oxide can form oxygen vacancy under the bombardment of high-energy electrons and carbon atoms and take advantage of the particularly active characteristics of the defect itself so that carbon atoms can adsorb and fill the defect site, so as to convert tungsten oxide into tungsten carbide. This method is simple and can be prepared on a large scale, thus, in situ converting tungsten oxide into tungsten carbide structure. The catalyst used for hydrogen precipitation reaction is conducive to the improvement in electrocatalytic performance and has important significance in solving the current energy and environmental issues.

## 2. Results and Discussion

### 2.1. Characteristics of Tungsten Carbide/Tungsten Oxide Heterostructure Nanosheets

**Figure 1:**
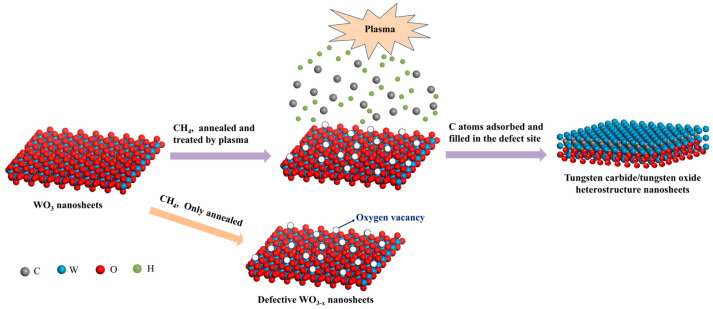
Preparation diagram of tungsten carbide/tungsten oxide heterostructure nanosheets.

Tungsten oxide powder was prepared through a hydrothermal reaction, and tungsten carbide/tungsten oxide heterostructure nanosheets were further prepared using plasma under a CH_4_ atmosphere. As shown in Figure 1, due to the reductive atmosphere of CH_4_, the defective tungsten oxide (WO_3-x_) can be constructed by reducing tungsten oxide (WO_3_) with CH_4_ at high temperatures. The tungsten oxide powder was placed in a tubular furnace at 750~850 °C, and the pressure was reduced to 20~40 Pa by vacuuming in a methane atmosphere. Meanwhile, the samples were focused with plasma for 60 min under the processing power of 200 W to obtain the tungsten carbide/tungsten oxide heterogeneous nanosheets denoted as WO3-x-750-P, WO3-x-800-P, and WO3-x-850-P, respectively. The surface of tungsten oxide can be converted into tungsten carbide in situ by filling the defect site of tungsten oxide with carbon atoms adsorbed, and the composite nanosheets of tungsten carbide/tungsten oxide can be realized finally. We modified the material structure by adjusting different plasma treatment temperatures, thus affecting the catalytic performance. Specifically, with the increase in plasma treatment temperature, the relative content of WC in the catalyst decreased, the relative content of W_2_C increased, and the corresponding activity also increased. It has also been reported that the dominant W_2_C phase exhibits a higher activity than WC since the free energy of hydrogen adsorption of W_2_C is close to zero [40].

**Figure 2:**
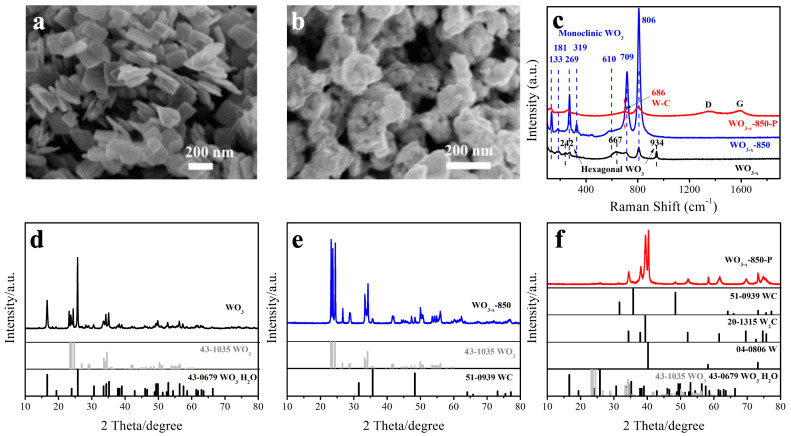
SEM images of WO_3_ (**a**), WO_3-x_-850-P (**b**), Raman and (**c**), XRD patterns of WO_3_ (**d**), WO_3-x_-850 (**e**), WO_3-x_-850-P (**f**), respectively.

The morphology and microstructure of these catalysts were observed by scanning electron microscopy (SEM). Figure 2a shows that the WO_3_ nanosheets synthesized by the hydrothermal method are rectangular nanosheets with a smooth surface and a particle size of around 200 nm. After annealing in the CH_4_ atmosphere, the WO_3-x_-850 particles are agglomerated to the coral-like network with smaller sizes of around 150–180 nm (Figure S3b). The morphologies for the samples annealed at different temperatures are shown in Figures S1–S3. It can be observed that the nanosheet agglomerates present an irregular shape with the increase in annealed temperature. After introducing the plasma to the annealing process in the CH_4_ atmosphere, the WO_3-x_-850-P sample shows a rough surface with wrinkles, still maintaining the network structure. Obviously, as the surficial morphology changes from smooth to rough, the specific surface area is enlarged (Figure 2b). As a result, there are more active sites involved in the electrochemical catalysis process. We also did the BET experiment (Figure S4); the specific area of the WO_3-x_-850-P increased to 24.21 m^2^ g^−1^ from 16.71 m^2^ g^−1^ of the WO_3_ sample. Similarly, we also explored the nanostructures obtained by plasma treatment at different temperatures under the CH_4_ atmosphere (Figures S5–S7). To confirm the elemental dispersion, the energy dispersive spectroscopy (EDS) mapping was also carried out, as shown in Figures S8–S10. There are almost no C signals on WO_3_ or WO_3-x_-850 samples, while the C signal is captured on WO_3-x_-850-P.

To further characterize the composition of the nanostructures, Raman spectroscopy (Figure 2c, Figures S11 and S12) is introduced to confirm the surface stretching modes of the obtained materials. The two peaks at 709 cm^−1^ and 806 cm^−1^ correspond to the O-W-O and W-O stretching models. The WO_3_ sample shows a mixture of hexagon and monoclinic WO structures [41]. By annealing at high temperatures without plasma, the dominant monoclinic WO phase was formed [41]. The WC stretching mode was also hardly found, which is also consistent with the above XRD data. After the induction of plasma in the CH4 atmosphere, an identical 686 cm^−1^ shift can be observed, indicating the formation of WC/W2C [40]. There are also D and G peaks on the WO_3-x_-850-P sample, which is also related to the existence of bulky C material [42]. It is also reported that the C impurity is inevitably formed in the tungsten carbide compound [42].

This result is consistent with X-ray diffraction (XRD) patterns. A very interesting phenomenon is found in the WO_3_ sample (Figure 2d), which consists of WO_3_ (PDF#43-1035) and WO_3_·H_2_O (PDF#43-0679). When the WO_3_ sample was annealed without plasma, only a trace amount of WC impurity was obtained (Figure 2e). As we introduced plasma to induce C doping (Figure 2f), the WC and W_2_C phases were obviously formed with the W impurity and residual WO_3_ (·H_2_O). The quantitative analysis shows that the material contains the dominant W_2_C components (76.519%). After plasma treatment, most of the tungsten oxide was converted to tungsten carbide. This transition indicates that the C doping hardly occurred directly under the CH_4_ atmosphere to form WC, while the plasma treatment induced to form the W_2_C phase. Moreover, with the increase in temperature, there is a structural transformation of WC to W_2_C in the structure of the material, mainly due to the instability of WC at high temperatures to decompose into W_2_C and C. Thus, it is observed that most of the components are W_2_C at 850 °C (Figures S13–S16). Interestingly, W_2_C has been reported in the literature as potentially more HER-active than WC [25].

**Figure 3:**
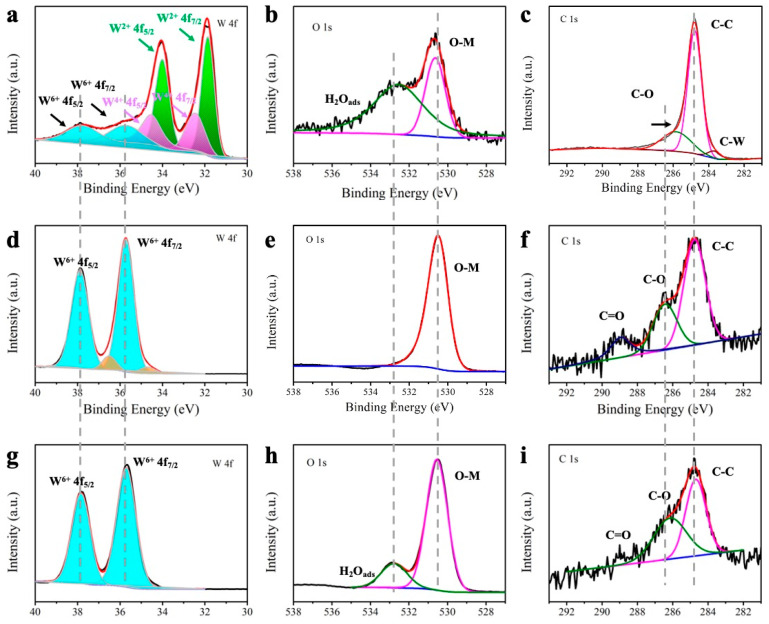
XPS spectra of (**a**,**d**,**g**) W 4f, (**b**,**e**,**h**) O 1s, and (**c**,**f**,**i**) C 1s for WO_3-x_-850-P, WO_3-x_-850, and WO_3_. Cyan, W^6+^; Orange, satellite peaks of W^6+^; Violet, W^4+^; Green, W^2+^.

X-ray photoelectron spectroscopy (XPS) was used to further study the electronic structure of synthetic materials. The XPS data of WO_3-x_-850-P (optimization in the later section), WO_3-x_-850, and WO_3_ are shown in Figure 3. In Figure 3a,d,g, the W 4f XPS treated using plasma in a methane atmosphere at 850 °C can be divided into three couples of subpeaks. The peaks at 31.64 eV and 33.74 eV can be attributed to W^2+^, indicating that W exists in the form of W_2_C [43,44]. The doublet peaks at 32.32 eV and 34.42 eV can be attributed to W^4+^, which are present in the form of WC (W 4f 7/2 and W 4f 5/2). The peaks at 35.75 eV and 37.85 eV are labeled as W^6+^, respectively, which corresponds to the residual compounds WO_3-x_ [43,44]. After plasma treatment, the peak area of tungsten oxide is significantly reduced, and a large area characteristic peak of W_2_C appears. The above results are exactly consistent with the previous XRD patterns, double-confirming that the C atom successfully occupies the defect position. In Figure 3b,e,h, the O 1s spectrum also indicates that a large number of H-O bonds are broken, and partial WO_3_ is converted into WO_3-x_ at the annealing temperature [45,46]. In Figure 3c,f,i, the C 1s peaks of WO_3_ and WO_3-x_ are fitted to three subpeaks at 284.7, 286.4, and 289.1 eV. The peak at 284.7 eV is assigned to (C-C/C=C), while the other two peaks are expressed as C-O and O=C-O existing on the surface of the adsorption, respectively. There is a subpeak fit on the WO_3-x_-850-P sample, indicating the formation of the C-W bond in WC/W_2_C [47].

### 2.2. Electrocatalytic HER Performance in 0.5 M Sulfuric Acid

**Figure 4:**
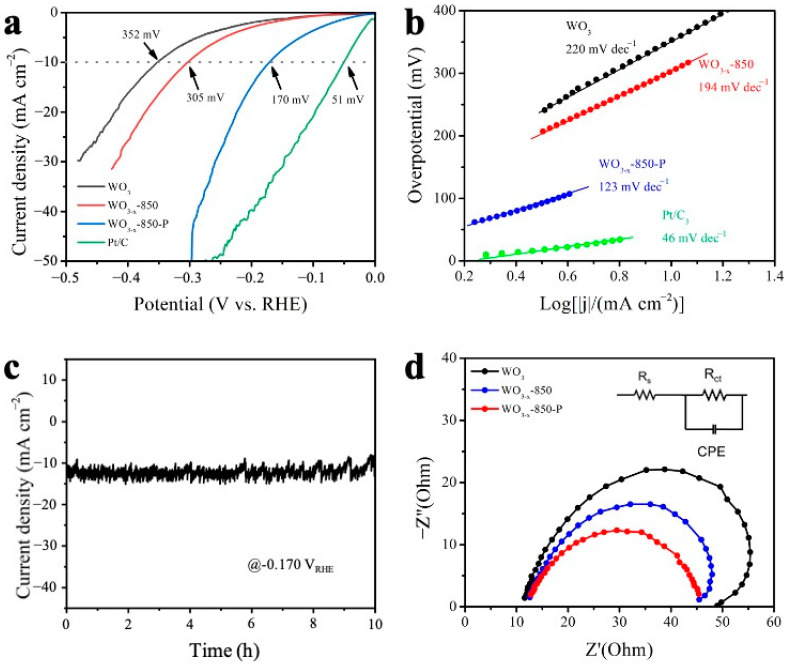
HER performance of WO_3-x_-850-P, WO_3-x_-850, and WO_3_ catalysts in 0.5 M H_2_SO_4_. (**a**) Polarization curves; (**b**) the corresponding Tafel slopes; (**c**) durability measurement of the WO_3-x_-850-P catalyst at the applied potential of −0.17 V vs. RHE; (**d**) Nyquist of WO_3_, WO_3-x_-850 and WO_3-x_-850-P.

The electrochemical polarization curves of the heterogeneous tungsten carbide catalyst, as well as the control samples for HER in 0.5 M H_2_SO_4_ electrolyte, are shown in Figure 4a,b. WO_3-x_-850-P exhibits the lowest overpotential of −170 mV vs. RHE to reach the current density of 10 mA cm^−2^. The optimization of the various temperatures with plasma is also shown in Figure S17, and it is found that 850 °C is the appropriate parameter to control the phase composition of WC and W_2_C content to improve electrochemical activity. With the increase in processing temperature, the relative content of WC in the catalyst is reduced, and the relative content of W_2_C increases, as well as the corresponding activity. It has also been reported the dominant W_2_C phase exhibits a higher activity than that of WC since the free energy of hydrogen adsorption of W_2_C is close to zero, although the WC phase exhibits excellent stability and electric conductivity in an electrochemical reaction [40]. Tafel slopes are also calculated to evaluate the HER Kinetics (Figure 4b). WO_3-x_-850-P has a low Tafel slope of 123 mV dec^−1^ compared with WO_3-x_-850 (194 mV dec^−1^) and WO_3_ (220 mV dec^−1^). The derived Tafel slope value of WO3-x-850-P is close to 120 mV dec^−1^, indicating that the rate-determining step on the catalyst surface is the Volmer step (H_2_O + e^−^ + M ⇌ M-H + OH^−^) [48]. This indicates that WO_3-x_-850-P accelerates the kinetics and the electron transfer process for HER. The comparison between the catalyst prepared in this work and the state-of-the-art tungsten carbide or noble-metal-based catalysts under similar conditions is shown in Table S1, exhibiting the enhanced HER activity in acidic media [11,49,50,51,52,53,54,55].

Durability is another critical parameter for evaluating the practical application of an HER electrocatalyst. A constant overpotential of −170 mV vs. RHE was applied on the WO_3-x_-850-P electrode while the output current density was maintained at −12 mA cm^−2^ for 10 h without degradation (Figure 4c), showing the stable durability for application. Compared with the original and defective tungsten oxide structures, the tungsten carbide and tungsten oxide nanostructures obtained after plasma treatment have better performance, which is mainly due to the metal-like properties of tungsten carbide and the heterogeneous structure between tungsten carbide and tungsten oxide, which can effectively improve the conductivity of the catalyst and form more active sites for hydrogen evolution reaction.

To elucidate the correlation between structural attributes and catalytic activity more profoundly, we employed in situ electrochemical impedance spectroscopy (EIS). This approach facilitated the establishment of relationships among interfacial kinetics, electron transfer, and mass transfer under varying potentials within electrochemical reactions [56]. The EIS plots and the simulated parameters are shown in Figure 4d and Table S2, respectively. The Rs represents the resistance between the working electrode to the reference, and the simulated values are around 11~12 ohms. The values of charge-transfer resistance (Rct) were also calculated from the fitting of the semicircles via an equivalent circuit model (Figure 4d, inset), reflecting the HER reaction rate. By comparing the diameter of the arc corresponding to the three samples at a specific potential, it can be concluded that the charge transfer resistance (Rct) of WO_3-x_-850-P is the smallest of merely 33 ohms, indicating that the catalyst involves a fast electron transfer process.

## 3. Materials and Methods

### 3.1. Preparation of WO_3_ Nanosheets

In total, 2.0 g Na_2_WO_4_·2H_2_O (AR, Sinopharm, China) and 1.2 g citric acid (AR, Sinopharm, China) were dissolved in 150 mL distilled water under vigorous stirring. Then, 6 M hydrochloric acid (36~38%, Sinopharm, China) aqueous solution was added to adjust the pH value to 1, and the light-yellow precipitate was obtained. Afterward, the suspension was transferred to a 200 mL stainless steel autoclave at 120 °C for 24 h. The products were separated by centrifugation at 9600 rpm and washed with distilled water and ethanol three times, respectively. The final products were dried in a vacuum drying oven for 12 h to obtain the WO_3_ nanosheets (denoted as WO_3_).

### 3.2. Preparation of WO_3-x_ Nanosheets

The massive WO_3_ nanosheets were directly treated in a methane atmosphere. To be specific, the tungsten oxide powder was placed in a tubular furnace at 750~850 °C, and the pressure was reduced to 20~40 Pa by vacuuming in the methane atmosphere. The obtained samples are denoted as WO_3-x-_750, WO_3-x_-800, and WO_3-x_-850, respectively.

### 3.3. Preparation of Tungsten Carbide/Tungsten Oxide Heterostructure Nanosheets

The massive WO_3_ nanosheets were treated at high temperatures using plasma technology in a methane atmosphere. To be specific, the tungsten oxide powder was placed in a tubular furnace (OTF-1200x, KJMTI, China) at 750~850 °C, and the pressure was reduced to 20~40 Pa by vacuuming in the methane atmosphere. Meanwhile, the samples were focused with plasma (elite 600, MKS, China) for 60 min under the processing power of 200 W to obtain the tungsten carbide/tungsten oxide heterogeneous nanosheets denoted as WO_3-x-_750-P, WO_3-x_-800-P, and WO_3-x_-850-P, respectively.

### 3.4. Electrochemical Measurement

In total, 4 mg catalyst was dispersed into 950 μL isopropyl alcohol under 10 min ultrasonic treatment, and then 50 μL Nafion (5wt%, Alfa, USA) was added to prepare the catalyst suspension under another 20 min ultrasonic treatment. 10 μL catalytic slurry was drop cast onto a glassy carbon electrode (5 mm diameter, GCE), and the electrode was air-dried at room temperature for two hours. The electrochemical performances were evaluated with Ivium potentiostat (Netherlands) in a three-electrode system, where the counter and the reference are graphite rod and saturated calomel electrode (SCE), respectively. All potentials versus an SCE electrode were converted to the potentials against the reversible hydrogen electrode (RHE) based on the Nernst equation: *E* (vs. RHE) = *E* (vs. SCE) + 0.0591pH + 0.242. All HER experiments were conducted in 0.5 M H_2_SO_4_ (pH = 1). The scan rate of LSV curves was 5 mV s^−1^. All data were not automatically corrected according to ohmic drop resistance. CVs were not proceeded before LSV scanning, but the LSV was repeated several times until the curves overlapped. EIS plots were performed under the applied potential −0.8 V vs. Ref. (−0.558 V vs. RHE) with an amplitude of 10 mV. The collected EIS plots were simulated by ZsimpWin 3.30d software by using the equivalent circuit of R_s_(QR_ct_).

### 3.5. Materials Characterization

The morphology and microstructure of the samples were investigated by scanning electron microscope (SEM, Hitachi, S-4800, Japan). The accelerating voltage is 5 kV, and the emission current is 10 μA. The X-ray diffraction (XRD, Rigaku D/MAX 2500, Japan) measurements used a diffractometer with Cu Kα radiation. The Raman spectra were collected on a Raman spectrometer (LabRAM-010, France) using a 532 nm laser. The X-ray photoelectron spectroscopy (XPS) measurements and analysis were recorded on an AXIS Supra (Axis Supra, Kratos, Japan), and the carbonaceous C1s line at 284.8 eV was used as a reference to calibrate the binding energies of other elements. The Brunauer–Emmett–Teller (BET) data of the obtained materials were obtained by a specific surface area analyzer (ASAP2020, USA).

## 4. Conclusions

In conclusion, tungsten carbide/tungsten oxide heterostructure nanosheets were successfully synthesized using a simple plasma method at 850 °C and under a CH_4_ atmosphere (named WO_3-x_-850-P). In an acidic medium, the synthesized WO_3-x_-850-P exhibits an enhanced HER performance of 10 mA cm^−2^ at the overpotential of −170 mV, which is lower than that of WO_3_ and WO_3-x_-850. In addition, the WO_3-x_-850-P catalyst also has a smaller Tafel slope, good stability, and a lower charge transfer resistance, thus showing better catalytic activity and electrical conductivity. In this study, a novel and efficient design method of tungsten carbide/tungsten oxide electrocatalysts was proposed, which provided a feasible way to explore more efficient tungsten-based electrocatalysts for electrocatalytic HER reactions.

## Data Availability

Data are contained within the article and Supplementary Materials.

## Supplementary Materials

The following supporting information can be downloaded at https://www.mdpi.com/article/10.3390/molecules30010084/s1. Figures S1–S3, SEM images of WO_3-x_-750, WO_3-x_-800, and WO_3-x_-850. Figure S4, BET of WO_3_ and WO_3-x_-850-P. Figures S5–S7, SEM images of WO_3-x_-750-P, WO_3-x_-800-P, and WO3-x-850-P. Figures S8–S10, EDS mapping of WO_3_, WO_3-x_-850, and WO3-x-850-P. Figure S11 and S12, Raman spectra. Figures S13–S16, XRD patterns. Figure S17, LSVs of the plasma-induced samples under various temperatures. Table S1, comparison of HER activity. Table S2, simulated parameters of EIS plots.
