# Supplementary material for: Tungsten Carbide/Tungsten Oxide Catalysts for Efficient Electrocatalytic Hydrogen Evolution
Source: Molecules. 2024 Dec 29;30(1):84. doi: 10.3390/molecules30010084 (PMC11720837; doi:10.3390/molecules30010084)
Supplement: Supplementary file 1 [file molecules-30-00084-s001.zip › molecules-3352329-supplementary.pdf]

Tungsten Carbide/Tungsten Oxide Catalysts for Efficient Electrocatalytic Hydrogen evolution

Jian Ouyang [1], Yu Sun [2,*], Yiqiong Zhang [3], Juzhe Liu [4], Xin Bo [2,*] and Zenglin Wang [2]

1 Shenzhen Kohodo Hydrogen Energy Co., Ltd., Shenzhen 518109, China; ouyangjian@kohodo.cn

2 Key Laboratory of Applied Surface and Colloid Chemistry, Ministry of Education, School of Chemistry and Chemical Engineering, Shaanxi Normal University, Xi'an 710119, China; sy15536162071@snnu.edu.cn (Y.S.)

3 College of Materials Science and Engineering, Changsha University of Science & Technology, Changsha, Hunan, 410114, China

4 Key Laboratory of Resources and Environmental Systems Optimization, Ministry of Education, College of Environmental Science and Engineering, North China Electric Power University, Beijing 102206, China

* Correspondence: sy15536162071@snnu.edu.cn (Y.S.); box@snnu.edu.cn (X.B.)

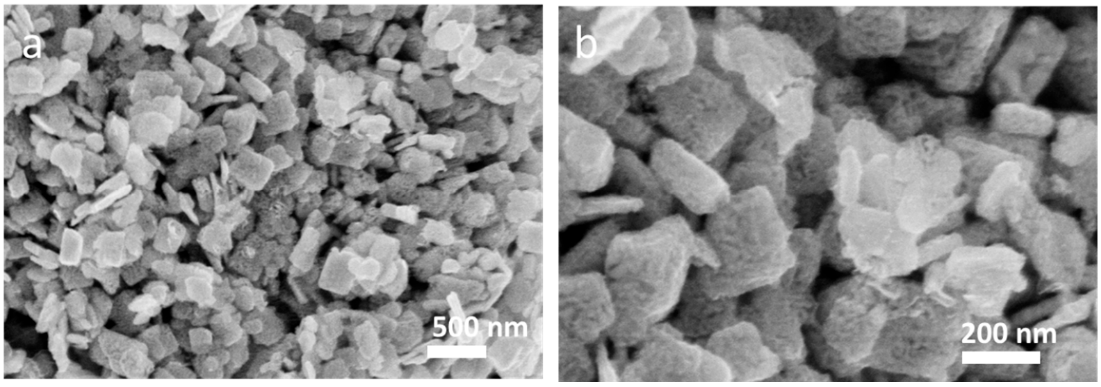

Figure S1. SEM images of the WO_{3-x}-750.

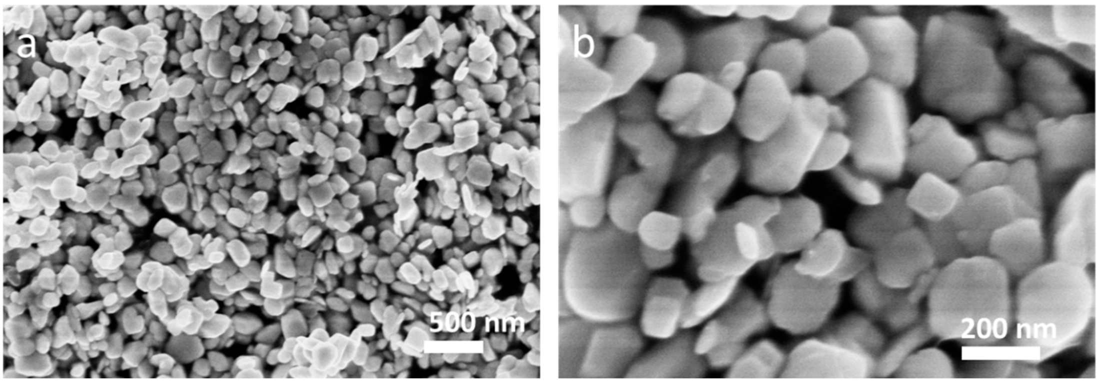

Figure S2. SEM images of the WO_{3-x}-800.

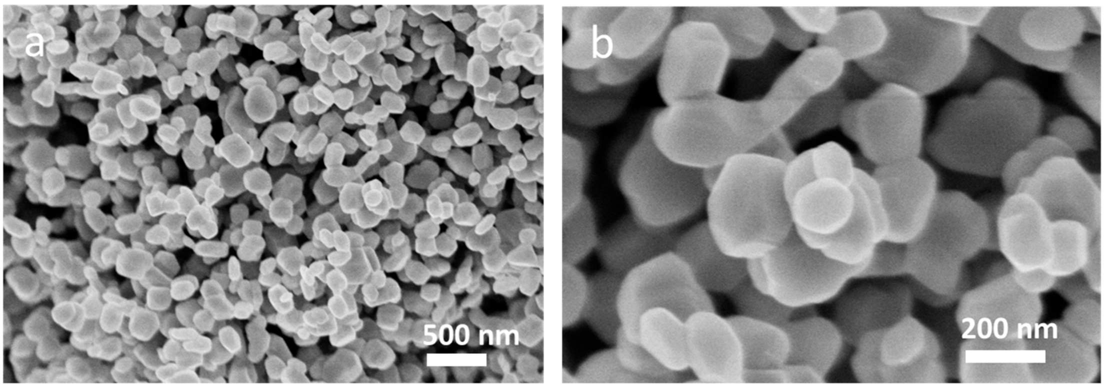

Figure S3. SEM images of the WO_{3-x}-850.

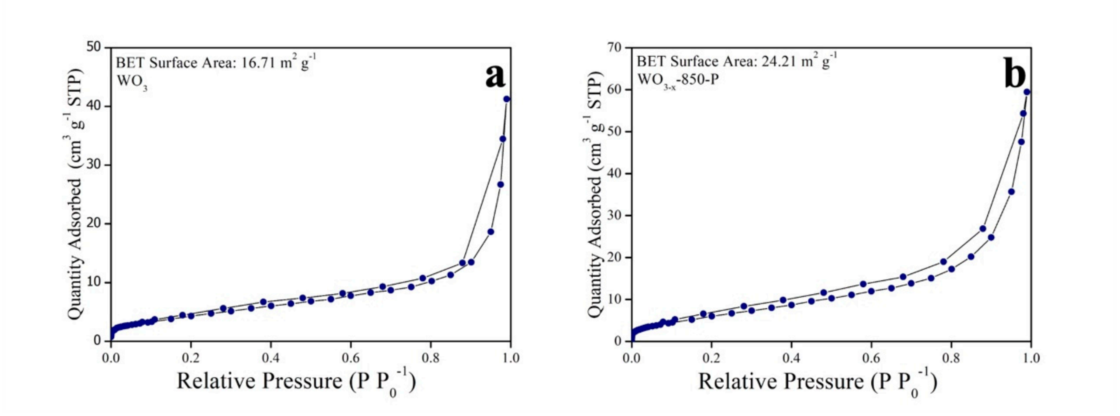

Figure S4. BET measurements of WO_3 (a) and WO_{3-x}-850-P (b)

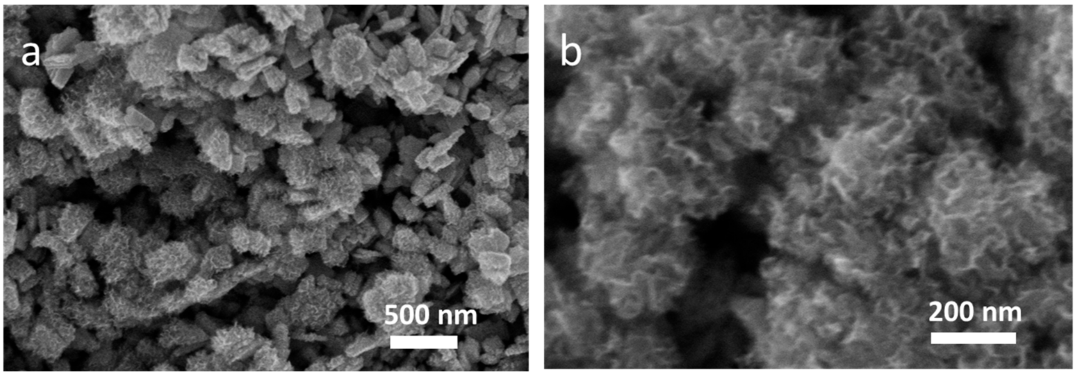

Figure S5. SEM images of the WO_{3-x}-750-P.

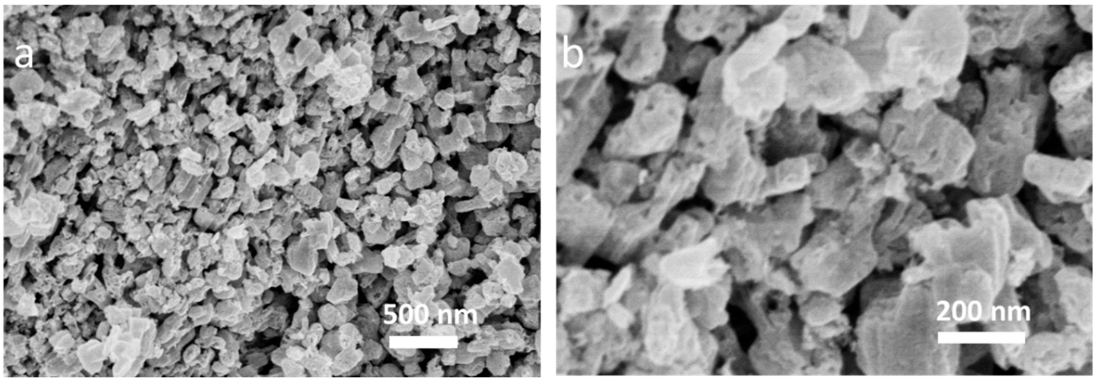

Figure S6. SEM images of the WO_{3-x}-800-P.

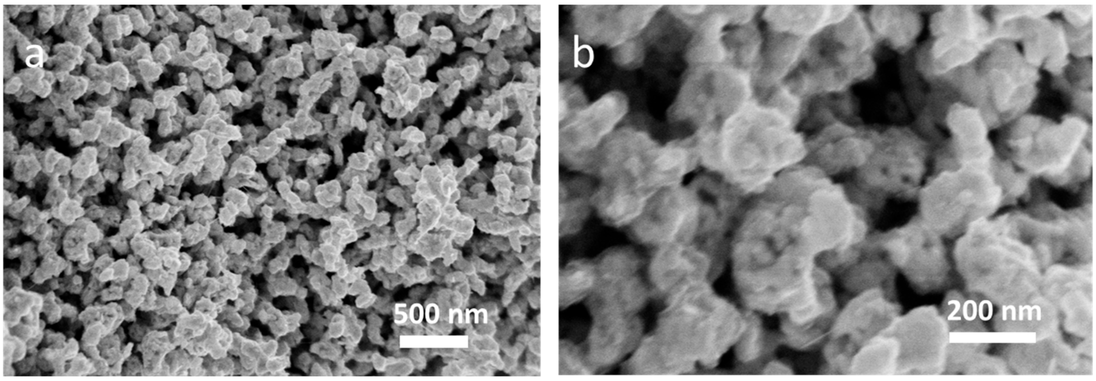

Figure S7. SEM images of the WO_{3-x}-850-P.

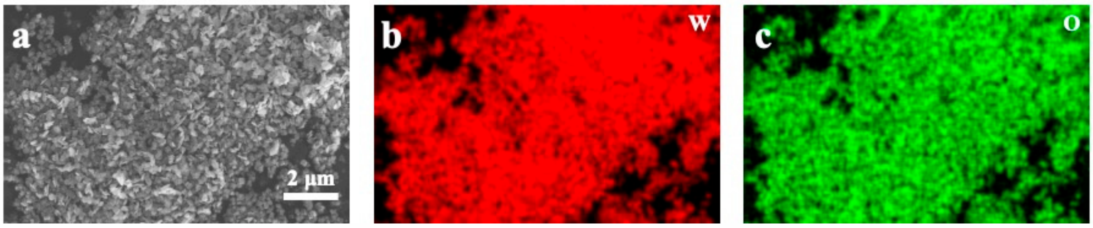

Figure S8. SEM (a) and the relevant EDS mapping of W (b) and O (c) elements on WO_3 sample.

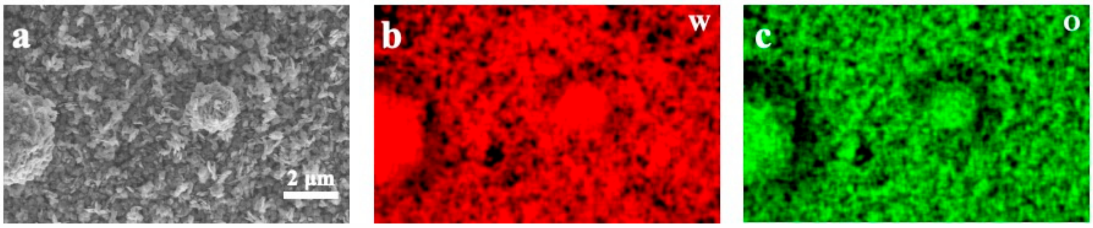

Figure S9. SEM (a) and the relevant EDS mapping of W (b) and O (c) elements on WO_{3-x}-850

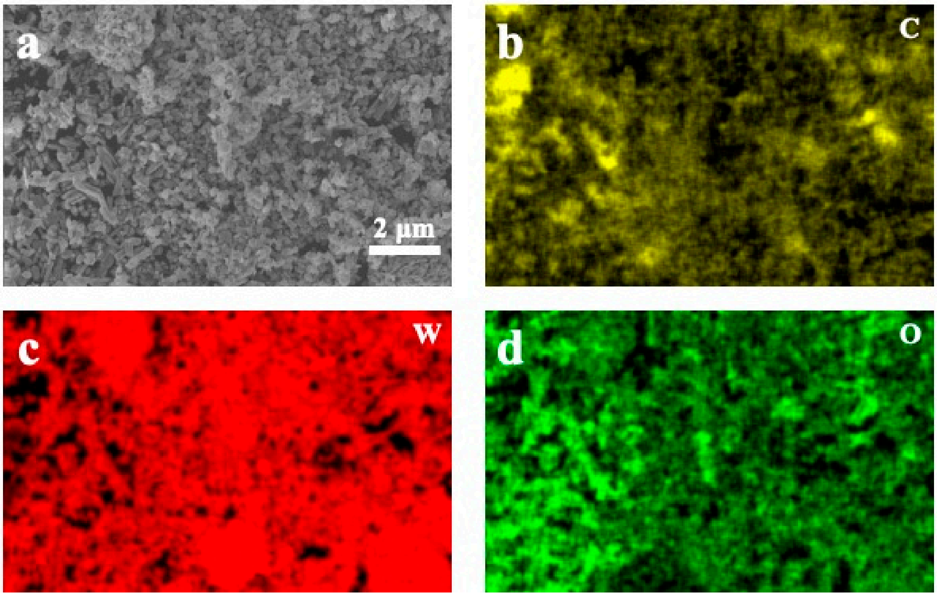

Figure S10. SEM (a) and the relevant EDS mapping of C (b), W (c) and O (d) elements on WO_{3-x}-850-P.

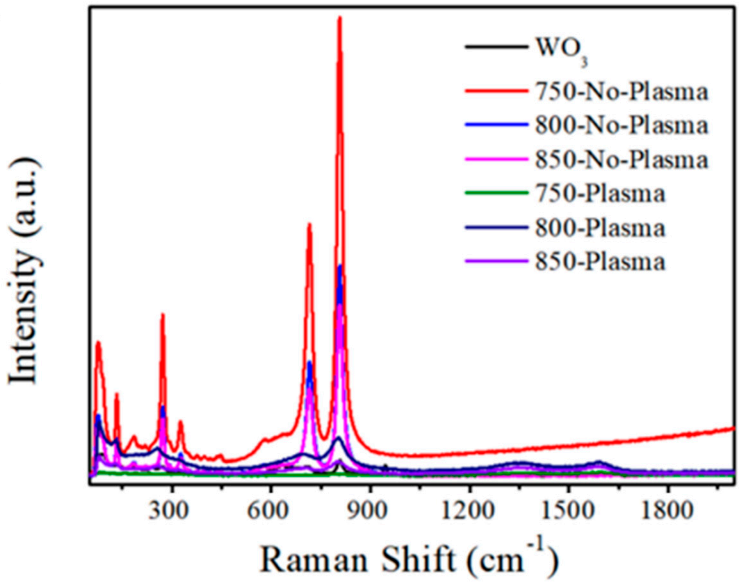

Figure S11. Raman spectra of WO_3 and WO_3 nanomaterials annealed at different temperatures or carburized at the same temperature and treated with plasma.

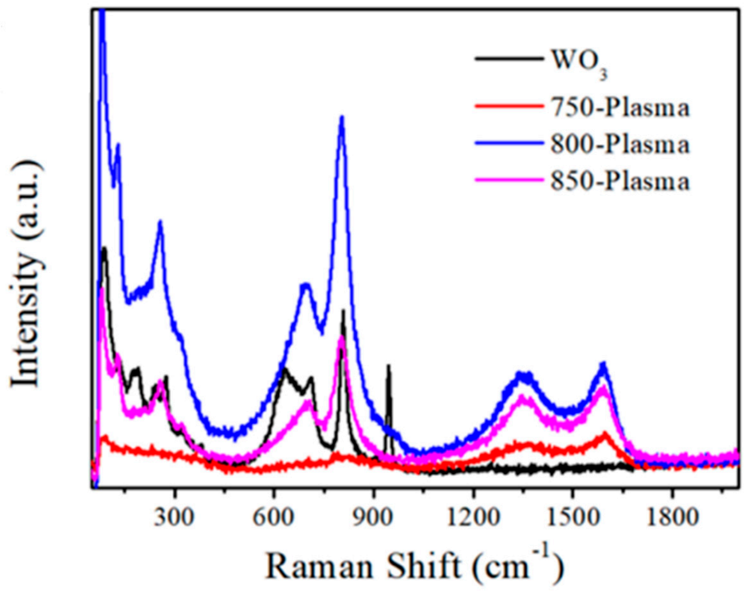

Figure S12. Raman spectra of WO_3 and WO_3 nanomaterials annealed at different temperatures and treated with plasma.

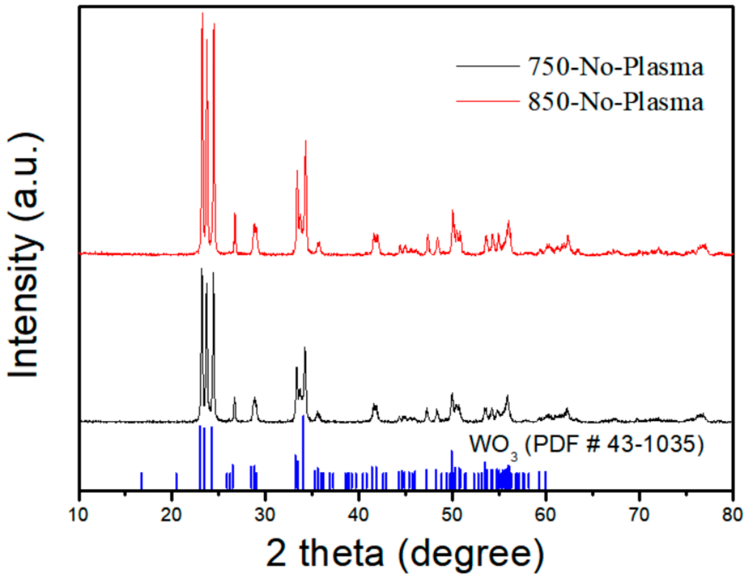

Figure S13. XRD patterns for the WO_{3-x}-750 and the WO_{3-x}-850.

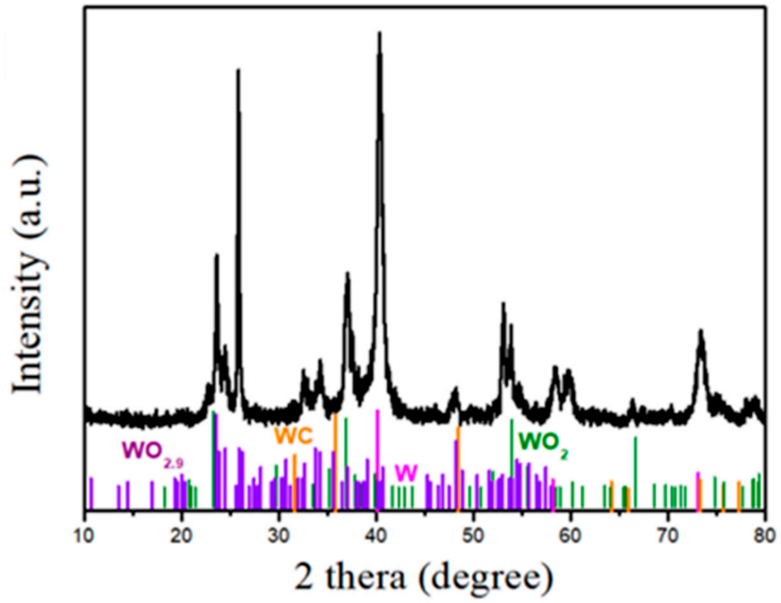

Figure S14. XRD patterns for the WO_{3-x}-750-P.

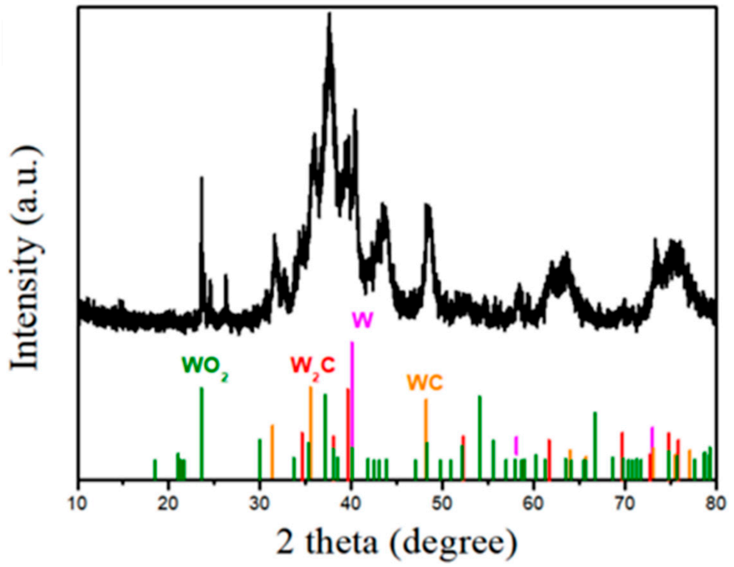

Figure S15. XRD patterns for the WO_{3-x}-800-P.

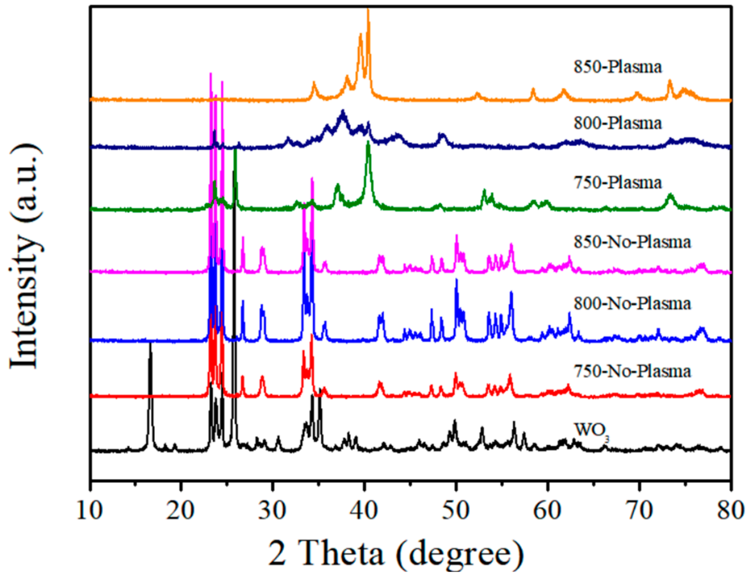

Figure S16. Comparative analysis of the XRD pattern for WO_3, heat treated with and without plasma under different temperature (750°C, 800°C, and 850°C).

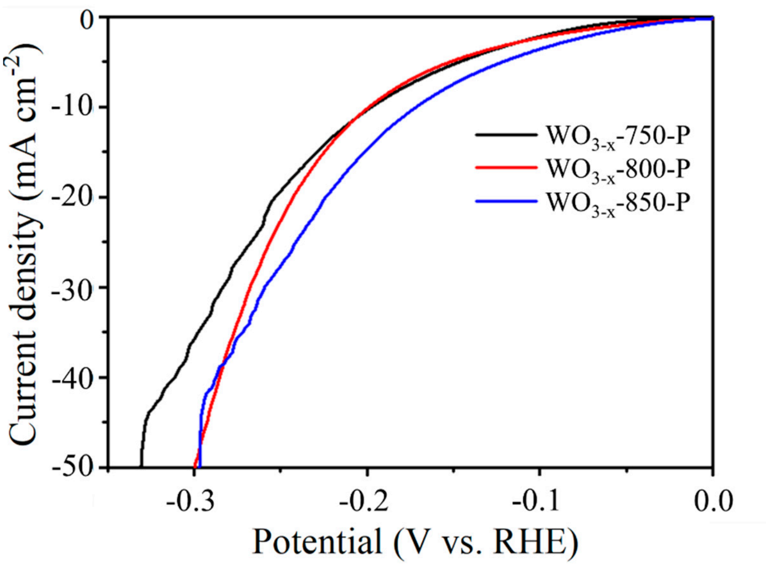

Figure S17. The LSV profiles of the WO_{3-x}-750-P, WO_{3-x}-800-P, and WO_{3-x}-850-P in 0.5 M H_2SO_4.

Table S1. HER activity comparison

electrolyte	Catalysts	Overpotential (mV) @10 mA cm-2	Ref.
0.5 M H_2SO_4	This work	170	This work
0.5 M H_2SO_4	Mo_2C/C hollow microspheres	175	[1]
0.5 M H_2SO_4	Mo_2CTx	189	[2]
0.5 M H_2SO_4	$Ta_{0.3}W_{0.7}C$/CB	~245	[3]
0.5 M H_2SO_4	Re@$Ti_3C_2T_x$	298	[4]
0.5 M H_2SO_4	WC_x/C	264	[5]
0.5 M H_2SO_4	N-doped WC	290	[6]
0.5 M H_2SO_4	W_2C-thinfilm	263	[7]
0.5 M H_2SO_4	WC/CNT	435	[8]

Table S2 Simulated parameters of EIS plots

Catalysts	R_s/Ohm	R_{ct}/Ohm	CPE/F	n
WO3	11.74	50.3	0.002593	0.8262
WO_{3-x}-850	12.63	38.71	0.002543	0.88942
WO_{3-x}-850-P	12.62	33.32	0.002998	0.89999

References

1. Wang, C.; Sun, L.; Zhang, F.; Wang, X.; Sun, Q.; Cheng, Y.; Wang, L., Formation of Mo-Polydopamine Hollow Spheres and Their Conversions to MoO_2/C and Mo_2C/C for Efficient Electrochemical Energy Storage and Catalyst. *Small* **2017**, 13, (32), 1701246.
2. Seh, Z. W.; Fredrickson, K. D.; Anasori, B.; Kibsgaard, J.; Strickler, A. L.; Lukatskaya, M. R.; Gogotsi, Y.; Jaramillo, T. F.; Vojvodic, A., Two-Dimensional Molybdenum Carbide (MXene) as an Efficient Electrocatalyst for Hydrogen Evolution. *ACS Energy Letters* **2016**, 1, (3), 589-594.
3. Hunt, S. T.; Kokumai, T. M.; Zanchet, D.; Román-Leshkov, Y., Alloying Tungsten Carbide Nanoparticles with Tantalum: Impact on Electrochemical Oxidation Resistance and Hydrogen Evolution Activity. *The Journal of Physical Chemistry C* **2015**, 119, (24), 13691-13699.
4. Suragtkhuu, S.; Sunderiya, S.; Purevdorj, S.; Bat-Erdene, M.; Sainbileg, B.; Hayashi, M.; Bati, A. S. R.; Shapter, J. G.; Davaasambuu, S.; Batmunkh, M., Rhenium Anchored $Ti_3C_2T_x$ (Mxene) Nanosheets for Electrocatalytic Hydrogen Production. *Nanoscale Adv* **2023**, 5, (2), 349-355.
5. Chen, Z.; Qin, M.; Chen, P.; Jia, B.; He, Q.; Qu, X., Tungsten

Carbide/Carbon Composite Synthesized by Combustion-Carbothermal Reduction Method as Electrocatalyst for Hydrogen Evolution Reaction. *International Journal of Hydrogen Energy* **2016,** 41, (30), 13005-13013.

6. Han, L.; Xu, M.; Han, Y.; Yu, Y.; Dong, S., Core-Shell-Structured Tungsten Carbide Encapsulated within Nitrogen-Doped Carbon Spheres for Enhanced Hydrogen Evolution. *ChemSusChem* **2016,** 9, (19), 2784-2787.
7. Zheng, H.; Huang, J.; Wang, W.; Ma, C., Preparation of Nano-Crystalline Tungsten Carbide Thin Film Electrode And Its Electrocatalytic Activity for Hydrogen Evolution. *Electrochemistry Communications* **2005,** 7, (10), 1045-1049.
8. Lin, J.-F.; Pitkänen, O.; Mäklin, J.; Puskas, R.; Kukovecz, A.; Dombovari, A.; Toth, G.; Kordas, K., Synthesis of Tungsten Carbide and Tungsten Disulfide on Vertically Aligned Multi-Walled Carbon Nanotube Forests and Their Application as Non-Pt Electrocatalysts for the Hydrogen Evolution Reaction. *Journal of Materials Chemistry A* **2015,** 3, (28), 14609-14616.
